# Molecular Mechanisms That Contribute to Horizontal Transfer of Plasmids by the Bacteriophage SPP1

**DOI:** 10.3389/fmicb.2017.01816

**Published:** 2017-09-22

**Authors:** Ana Valero-Rello, María López-Sanz, Alvaro Quevedo-Olmos, Alexei Sorokin, Silvia Ayora

**Affiliations:** ^1^Department of Microbial Biotechnology, Centro Nacional de Biotecnología, Consejo Superior de Investigaciones Científicas Madrid, Spain; ^2^Micalis Institute, INRA, AgroParisTech, Universite Paris-Saclay Jouy-en-Josas, France

**Keywords:** horizontal gene transfer, plasmid transduction, SPP1, bacteriophages, antibiotic resistance

## Abstract

Natural transformation and viral-mediated transduction are the main avenues of horizontal gene transfer in Firmicutes. *Bacillus subtilis* SPP1 is a generalized transducing bacteriophage. Using this lytic phage as a model, we have analyzed how viral replication and recombination systems contribute to the transfer of plasmid-borne antibiotic resistances. Phage SPP1 DNA replication relies on essential phage-encoded replisome organizer (G*38*P), helicase loader (G*39*P), hexameric replicative helicase (G*40*P), recombinase (G*35*P) and in less extent on the partially dispensable 5′→3′ exonuclease (G*34*.*1*P), the single-stranded DNA binding protein (G*36*P) and the Holliday junction resolvase (G*44*P). Correspondingly, the accumulation of linear concatemeric plasmid DNA, and the formation of transducing particles were blocked in the absence of G*35*P, G*38*P, G*39*P, and G*40*P, greatly reduced in the G*34*.*1*P, G*36*P mutants, and slightly reduced in G*44*P mutants. In contrast, establishment of injected linear plasmid DNA in the recipient host was independent of viral-encoded functions. DNA homology between SPP1 and the plasmid, rather than a viral packaging signal, enhanced the accumulation of packagable plasmid DNA. The transfer efficiency was also dependent on plasmid copy number, and rolling-circle plasmids were encapsidated at higher frequencies than theta-type replicating plasmids.

## Introduction

Bacteriophage-mediated horizontal gene transfer enhances bacterial adaptive responses to environmental changes, and it is one of the mechanisms responsible for the rapid spread of antibiotic resistance, bacterial virulence and pathogenicity (Canchaya et al., 2003; Brussow et al., 2004; Brown-Jaque et al., 2015; Penades et al., 2015; Touchon et al., 2017). Bacteriophages, or simply phages, play active roles in the specialized mobilization of discrete chromosomal regions (specialized transduction), and also with significant efficiency can transfer any chromosomal segment or plasmid DNA (generalized transduction). The difference between these two transduction modes is that specialized transduction is the consequence of the faulty excision of the prophage from the bacterial chromosome, resulting into packaging of phage DNA as well as adjacent DNA from the bacterial chromosome (Canchaya et al., 2003; Brussow et al., 2004; Penades et al., 2015; Touchon et al., 2017). In generalized transduction, phage DNA mispackaging occurs, and the viral packaging machinery uses chromosomal or plasmid DNA as a substrate for DNA packaging into the empty proheads instead of viral DNA (Ikeda and Tomizawa, 1965; Viret et al., 1991). Generalized transduction, which is recognized as a widespread mechanism for the transfer of any gene from one bacterium to another, was originally reported in γ-proteobacteria (Zinder and Lederberg, 1952; Lennox, 1955), and it has been also reported in many Gram-positive pathogens (Maslanova et al., 2013; Giovanetti et al., 2014; Winstel et al., 2015). The majority of generalized transducing phages package their DNA by the headful packaging mechanism (*pac* phages). One remarkable event related to this, is the encapsidation of pathogenicity islands, as it occurs with the *Staphylococcus aureus* pathogenicity islands (SaPIs). SAPIs have developed elegant strategies to hijack the phage machinery to use it for their own transfer (Penades et al., 2015). Most SaPI helper phages identified to date are *pac* phages, and many well-studied SaPIs are packaged by the headful mechanism (Ruzin et al., 2001). Despite its importance in spreading antibiotic resistances and virulence, the mechanisms that occur inside the cell and lead to the erroneous encapsidation of foreign DNA upon phage infection remain largely unexplored.

SPP1 is a 44-kb virulent *Bacillus subtilis* phage that can carry out generalized transduction (plasmid and chromosomal) with a significant frequency (Yasbin and Young, 1974; Ferrari et al., 1978; Canosi et al., 1982). The SPP1 replication and packaging machineries have been studied in deep (Alonso et al., 2006; Lo Piano et al., 2011; Oliveira et al., 2013). SPP1 DNA replication starts by the theta mode when the replisome organizer, G*38*P, binds to the replication origin, *ori*L (Pedre et al., 1994; Missich et al., 1997; Seco and Ayora, 2017). Then, the phage helicase loader (G*39*P) recruits the replicative haxameric helicase (G*40*P). The viral helicase recruits the host-encoded primase (DnaG) and DnaX, which is a subunit of the clamp loader (Pedre et al., 1994; Ayora et al., 1999; Martinez-Jimenez et al., 2002), so that a full replisome is loaded at the phage origin. SPP1 replication uses the host replicase holoenzyme and topoisomerases from the host (Seco et al., 2013; Seco and Ayora, 2017). After one or two rounds of theta-type replication (TR), it shifts to concatemeric (sigma-type) DNA replication in a process driven by recombination (Lo Piano et al., 2011). Two viral proteins may participate in this shift, the ATP-independent single-strand annealing recombinase (G*35*P) and its partner, the 5′→3′ exonuclease (G*34*.*1*P) (Ayora et al., 2002; Martinez-Jimenez et al., 2005). In the shift to concatemeric DNA replication, G*38*P, bound to *ori*R, or working as a pre-primosome organizer (like the bacterial PriA enzyme), may restart DNA replication at stalled or paused replication forks (Seco et al., 2013). SPP1 codes for two other proteins involved in DNA replication and recombination: the G*36*P and G*44*P proteins. G*36*P is a single-stranded DNA binding protein (SSB), and G*44*P is a Holliday junction resolvase of the RusA family, which recognizes and cleaves a variety of recombination intermediates (Martinez-Jimenez et al., 2005; Zecchi et al., 2012). Biochemical assays showed that G*36*P is crucial for SPP1 DNA replication *in vitro*, but it can be substituted by host-encoded SSB (known as SsbA) (Seco et al., 2013). The role of G*44*P in SPP1 replication is thought to be the processing of the stalled replication fork, which may trigger the shift to the sigma-type or concatemeric DNA replication. This type of DNA replication is essential to generate the concatemeric DNA, which is the substrate for encapsidation. Viral replication and packaging are sequential and in some way coupled events. SPP1 encapsidates linear double-strand (ds) DNA into an empty prohead by a processive (∼4 sequential packaging cycles) headful packaging mechanism, using the linear head-to-tail concatemer as a substrate (Oliveira et al., 2013). This is consistent with the observation that an *in vitro* DNA packaging system efficiently packaged mature SPP1 DNA as well as linear plasmid DNA, but no DNA packaging could be detected when circular DNA was the substrate for encapsidation (Oliveira et al., 2005). SPP1 packaging is initiated with the recognition of the specific *pac* region by the terminase small subunit, G*1*P, and the sequence specific cleavage at the *pac* sequence (CTATTGCGG↓C) by the terminase large subunit, G*2*P (Chai et al., 1992, 1995, 1997). This generates the first DNA end to be encapsidated (Chai et al., 1992; Gual et al., 2000; Camacho et al., 2003). A sequence independent cleavage, at 104% of the genome (headful cleavage), terminates one packaging round, generating a new starting point for another one (Chai et al., 1995; Camacho et al., 2003). Hence, the first cleavage in the concatemeric SPP1 DNA occurs specifically at *pac*, whereas the next ones do not (Gual et al., 2000).

In addition to package viral DNA, SPP1 is able to encapsidate chromosomal or plasmid DNA. However, some differences were observed with these two substrates. Rolling-circle replicating plasmids could be transduced at a frequency much higher than chromosomal DNA (Ferrari et al., 1978; Deichelbohrer et al., 1985), and an explanation for this could be that the copy number of plasmids in the cell is higher than that of the chromosome. Alternatively, another possibility could be that the replication mode influences the transduction frequency. It was also observed that the frequency of transduction of pUB110 and pC194 naturally occurring plasmids was enhanced 100- to 1000-fold by the presence of inserts homologous to the transducing phage DNA (Deichelbohrer et al., 1985). This homology-facilitated plasmid transduction was independent of the host RecA (Canosi et al., 1982; Deichelbohrer et al., 1985). In contrast, another report showed that SPP1 mediated chromosomal transduction was reduced 30-fold in cells having mutations in host functions involved in homologous recombination, such as RecA, RecU, and RecF (Ferrari et al., 1978). These differences, which were observed between plasmid and chromosomal transduction in the SPP1 system motivated us to analyze in deep and throughout the manuscript the influence of the replication mode and of the plasmid copy number in plasmid generalized transduction. In addition, we have analyzed the role of phage recombination and replication proteins. We show that in absence of G*35*P, G*38*P, G*39*P, or G*40*P linear plasmid transduction is blocked. In contrast, establishment of injected linear plasmid DNA in the recipient host was independent of viral-encoded functions. The transfer efficiency was found to be dependent on homology to phage DNA, plasmid copy number, and replication mechanism.

## Materials and Methods

### Bacterial Strains and Plasmids

*Bacillus subtilis* BG214 (*trpCE metA*5 *amyE1 ytsJ*1 *rsbV*37 *xre*1 *xkd*A1 *att*^SPß^ *att*^ICE^*^*Bs1*^*) and its isogenic derivative BG295 (*sup*3) were used. They lack the ICE*Bs1* integrative conjugative element as well as prophage PBSX, and PBSX prohage cannot be induced (Kidane et al., 2009). The plasmids used are derivatives of pHP13, pUB110, pBT233 or pNDH33 (**Table 1**). To construct pBT233N, the pUB110 neomycin resistance gene was cloned into AvaI-linearized pBT233. Different regions of the SPP1 genome were cloned into the HpaI site of the pBT233N plasmid as indicated in **Table 1**. pHP13 derivatives were kindly provided by J. C. Alonso (CNB-CSIC). Plasmid pBT400 is a pHP13 derivative bearing an EcoRI-SalI fragment of SPP1 DNA. Different SPP1 DNA fragments were cloned into XbaI- or SmaI-cleaved pNDH33 DNA, rendering pNDH33-1300 and pNDH33-*pac* (**Table 1**).

**Table 1:** Plasmids used in this work.

Plasmids	Plasmid characteristics	Reference
pC194	Natural rolling circle replicating (RCR) plasmid, 2.9-kb	Horinouchi and Weisblum, 1982; Alonso and Trautner, 1985
pHP13	RCR plasmid derivative of pTA1060, 4.9-kb	Haima et al., 1987
pBT163 (pHP13-*pac*)	pHP13 derivative containing SPP1 DNA including *pac* (2675 bp cloned, coordinates 43778–44010 and 1–2439)	Chai et al., 1992
pBT271 (pHP13-*ori*L*)*	pHP13 derivative containing SPP1 DNA including *ori*L (2975 bp, coordinates 33875–36850)	Chai et al., 1993
pBT400 (pHP13-800)	pHP13 derivative containing SPP1 DNA (864 bp, coordinates 3225–4089)	This work
pUB110	Natural RCR plasmid, 4.5-kb	Leonhardt, 1990
pUB110-cop1	pUB110 derivative, lower copy number	Leonhardt, 1990
pBG55 (pUB110-3600)	pUB110 derivative containing SPP1 DNA (3639 bp, coordinates 23117–26756)	Deichelbohrer et al., 1985
pBT233	Theta replicating (TR) plasmid, 9-kb	Ceglowski et al., 1993a
pBT233N	pBT233 derivative containing the 1304 bp neomycin resistance gene (N) from pUB110	This work
pBT233N-400	pBT233N derivative containing SPP1 DNA (414 bp, coordinates 32562–32976)	This work
pBT233N-1300	pBT233N derivative containing SPP1 DNA (1340 bp, coordinates 25051–26391)	This work
pBT233N-*ori*L	pBT233N derivative containing SPP1 *ori*L DNA (350 bp, coordinates 35801–36151	This work
pBT233N-*pac*	pBT233N derivative containing SPP1 *pac* DNA (412 bp, coordinates 43689–44010 and 1–70)	This work
pNDH33	TR plasmid derivative of pBS72, 8.1-kb	Titok et al., 2003
pNDH33-1300	pNDH33 derivative containing SPP1 DNA (1340 bp, coordinates 25051–26391)	This work
pNDH33-*pac*	pNDH33 derivative containing SPP1 *pac* DNA (412 bp, coordinates 43689–44010 and 1–70)	This work

### SPP1 Phages

The SPP1 phages used in this work are listed in **Table 2**, including those (*sus19, sus53, sus109*, tsB3, and SPP1ΔA) previously described (Chai et al., 1992; Pedre et al., 1994; Zecchi et al., 2012).

**Table 2:** SPP1 phages used in this work.

Genotype	Name	Activity / type of mutant	Reference
wt	SPP1wt	Wild type	
*34*.*1*^-^	*sus34.1*	Exonuclease, ochre mutant (OM)	This work
*35*^-^	*sus35*	Recombinase, OM	This work
*35*^-^	tsI20F	Recombinase/thermosensitive (ts) mutant	This work
*36*^-^	*sus36*	ssDNA binding protein, OM	This work
*38*^-^	tsB3	Replisome organizer/ts mutant	Pedre et al., 1994
*39*^-^	*sus53*	Helicase loader, OM	Pedre et al., 1994
*40*^-^	*sus109*	Helicase, OM	Pedre et al., 1994
*44*^-^	SPP1ΔA	Deletion mutant lacking Holliday junction resolvase	Zecchi et al., 2012
*2*^-^	*sus19*	Terminase large subunit, OM	Chai et al., 1992

The SPP1 tsI20F mutant was sequenced and it was found that the mutation that conferred thermosensitivity (ts), P159S, mapped in gene *35*, rather than in gene *34.1*, as it was previously suggested after genetic mapping (Burger and Trautner, 1978). This phage was used to construct the SPP1 *sus35* mutant. First, a lysine codon (the 10th codon in the gene *35*) was replaced by an ochre (UAA) stop codon by site-directed mutagenesis using plasmid pCB610 as template (a pHP13 derivative containing SPP1 genes *34.4* to *35*) and the Quickchange protocol. After sequencing confirmation the resulting plasmid (pHP13-G*35*P-ochre) was introduced into BG295 cells by transformation. BG295 cells bearing pHP13-G*35*P-ochre plasmid were infected with SPP1 tsI20F phage at 30°C for 2 h. The resulting phage lysate was used to infect BG295 cells at non-permissive temperature to obtain the recombinant phages. They were picked from Luria-Bertani (LB) plates supplemented with 10 mM MgCl_2_ (LB-Mg^+^) incubated at 50°C. The amplified phage was sequenced to confirm that phages had acquired the ochre mutation in gene *35*, and that it had reverted to wt the tsI20F mutation. The resulting mutant phage, containing the ochre codon, was named SPP1 *sus35*.

The 37th codon (Lys) in gene *36* was replaced by an ochre (UAA) stop codon in a pHP13 derivative containing SPP1 genes *34.4* to *37*. The SPP1 *sus34.1* mutant was generated by replacing, in a pHP13 derivative containing SPP1 genes *34.1* to *35*, the 31th codon (AAA) of gene *34.1* by an ochre (UAA) stop codon. The SPP1 *sus36* and *sus*34.1 mutants were then generated by homologous recombination between the SPP1 tsI20F phage and these plasmids carrying the stop ochre codon into the gene to be mutated, as described above. The accuracy of the resulting mutant phages was confirmed by sequencing.

SPP1wt, SPP1ΔA phages and the thermosensitive phages (tsI20F, and tsB3) were amplified in BG214 cells grown at 37°C or 30°C in LB-Mg^+^, whereas the *sus* phages were routinely amplified in the suppressor strain BG295 (*sup3*) at 37°C.

### Preparation of Transducing Lysates

Transducing lysates were obtained by infecting with the different SPP1 phages, at a multiplicity of infection (MOI) of 10, *B. subtilis* BG214 cells bearing the indicated plasmids, grown up to mid-exponential phase in LB-Mg^+^ and appropriated antibiotics. Aliquots were taken at different post-infection times for DNA analysis and processed as described below. The cultures were centrifuged after 90 min of infection (14,000 rpm, 5 min), and the supernatants were filtered through 0.45 μm filters to remove donor cells. Under these growth conditions *B. subtilis* cells are not competent, so that DNAse I treatment was not required. Phage lysates were titrated on BG214 cells or BG295 cells before use and were stored at 4°C.

### Plasmid Transduction

Exponentially growing recipient *B. subtilis* BG214 or BG295 cells (OD_560_ = 0.4) grown at 37°C in LB-Mg^+^, were infected with the transducing phage lysate at MOI of 1. Phages were allowed to be absorbed for 5 min, and then the non-absorbed phages were removed by centrifugation. Cell pellets were washed and finally resuspended in 1 ml LB. Appropriate dilutions were plated in selective LB-agar plates containing the respective antibiotics, and incubated overnight at 37°C to quantify the number of transductants. As a control, 1 ml of the recipient host was plated to discard the appearance of spontaneous resistant colonies. In another LB-agar plate with antibiotic the same amount of the stock transducing lysate was plated without recipient cells, to discard a contamination with donor cells.

### Analysis of Plasmid DNA Forms

*B. subtilis* BG214 cells bearing the different plasmids were grown at 37°C to an OD_560_ of 0.40 in LB-Mg^+^ media supplemented with appropriate antibiotics, and infected with a MOI of 10. Phage addition marked the time zero of our experiments. At given times, aliquots of 1ml were collected, rapidly placed in a water-ice mixture and centrifuged for 5 min at 14,000 rpm and 4°C. The pellets were stored at -80°C. In experiments with thermosensitive phage mutants, the strains bearing plasmids were first grown at 30°C to an OD_560_ of 0.2, transferred to 50°C and then further grown to OD_560_ of 0.4. They were infected at 50°C, and the samples were processed as described above. Total DNA was isolated following a protocol described earlier (Viret and Alonso, 1987) with some minor modifications. Samples were resuspended in 200 μl of lysis buffer (25 mM Tris-HCl pH 8.0, 50mM glucose, 10 mM EDTA, 0.5 mg/ml lysozyme and 0.1 mg/ml RNase A). After 30 min of incubation at 30°C, Proteinase K (0.5 mg/ml) and SDS (0.8%) were added, and the mixture was further incubated for 30 min at 37°C. The lysate obtained was then treated twice with phenol and dialyzed against 20 mM Tris-HCl pH 8.0, 1 mM EDTA.

Pulsed field gel electrophoresis (PFGE) was performed on a Bio-Rad CHEF-DR II apparatus. 15 μl of samples were loaded on the 1% agarose gel. Running conditions were 5 V/cm, 0.5% TBE, 0.5–10 switch time for 20 h at 14°C. The molecular weight marker used was LW range PFG marker or λ DNA-HindIII digest, both from New England Biolabs. The probe used for Southern blot hybridization was a PCR product of 500 bp corresponding to neomycin or chloramphenicol resistance genes. Southern blots were performed with Hybond-N+ membranes as recommended by the manufacturer (GE Healthcare), and detection was done with the AlkPhos Direct Labeling kit (GE Healthcare).

## Results

### Viral Replication and Recombination Proteins Are Responsible for the Generation of Plasmid Transducing Particles

To unravel the mechanisms that contribute to SPP1-mediated horizontal plasmid transfer we used *B. subtilis* BG214 strain, which is non-inducible for PBSX prophage and lacks prophage SPβ and the ICE*Bs1* integrative conjugative element. To analyze the role in antibiotic resistance transfer of SPP1 replication and recombination proteins, phages *sus34.1* and *sus36*, bearing mutations in genes *34.1* and *36* respectively, were constructed. SPP1 phage variants bearing mutations in the other genes were available in our phage collection (*sus19, sus53, sus109*, SPP1ΔA, tsB3). For comparison, a SPP1 *sus35* phage was also constructed, although a thermosensitive gene *35* mutant (the tsI20F phage) was available. The list of the bacteriophages used is shown in **Table 2**.

**FIGURE 1:**
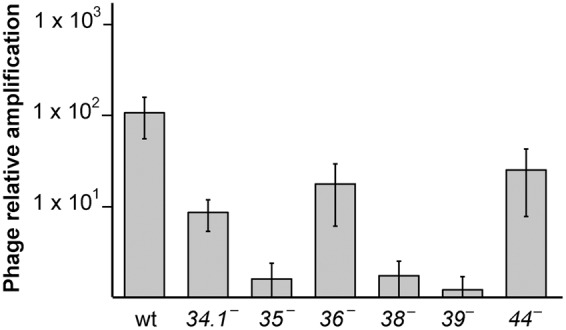
Effect of the different SPP1 mutations on phage titer. *B. subtilis* BG214 cells were infected with the different phages at a MOI of 10, and after 2 h of infection PFU/ml was calculated. The number of phages, relative to the number of phages initially added, is indicated. The values are the mean of at least five independent assays and error bars indicate SD.

First we analyzed if G*34*.*1*P and G*36*P proteins, which were not yet studied *in vivo*, are essential for SPP1 replication (**Figure 1**). BG214 cells were grown until mid-exponential phase and then infected at MOI of 10 with the SPP1wt, SPP1ΔA, tsB3 (at restrictive temperature), or the different *sus* mutants (*sus34.1, sus35, sus36*, and *sus53*, a phage with a mutation in gene *39*). After 90 min of infection, the phage lysates were collected and titrated. As previously observed, deletion of gene *44* reduced the phage titer only 5-fold (Zecchi et al., 2012), whereas the mutation in gene *35, 38* or *39* completely abolished SPP1 amplification (Pedre et al., 1994; Ayora et al., 2002). The mutation in gene *36* reduced SPP1 titer only 6-fold, in agreement with the biochemical data showing that G*36*P can be replaced by the host SsbA during SPP1 DNA replication (Seco et al., 2013; Seco and Ayora, 2017). Deletion of the *34*.*1* gene reduced the phage titer 10-fold, and the size of the phage plaques was considerably smaller compared to the wt phage (Supplementary Figure S1). These results show that both, G*36*P, and G*34*.*1*P are not essential for phage amplification, although their defects reduce phage development.

**FIGURE 2:**
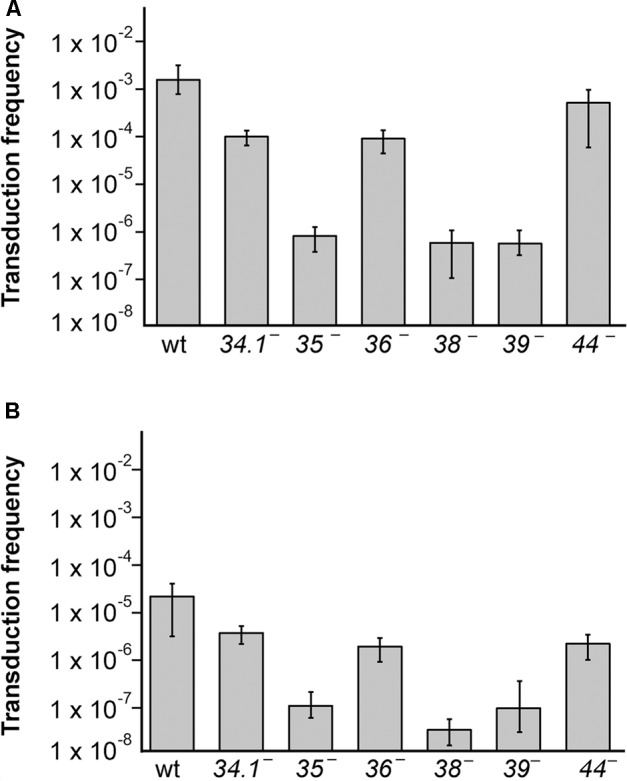
The generation of transducing lysates bearing plasmids with (pBG55) or without (pUB110) sequence homology with SPP1 is affected by mutations in viral replication and recombination genes. **(A)** Generation of pBG55 transducing particles after phage infections, expressed as frequency of transductants/CFU. **(B)** Generation of pUB110 transducing particles. BG214 cells bearing plasmids were infected with the different phage mutants and lysates were used to infect the BG295 *sup3* strain to have the *sus* mutation only in the donor cells. The values are the mean of at least four independent assays and error bars indicate SD.

To analyze if SPP1 replication and recombination proteins are involved in the generation of the transducing particle, the different *sus* mutant phages were used to infect BG214 cells bearing plasmid pBG55, a rolling circle replicating (RCR) plasmid with high-frequency of transduction (see **Table 1** for more description). The lysates were collected after 90 min of infection, filtered and used to infect the BG295 *sup3* strain, to have the effect of phage *sus* mutation only in the donor and not in the recipient strain. The frequency of pBG55 transfer (Neomycin resistants [Nm^R^]/CFU) for the wt phage was similar to previously published results obtained using the BG214 strain, both as donor and as recipient (Deichelbohrer et al., 1985). These results show that the *sup3* genotype does not affect the transduction frequency. In parallel, infections with the thermosensitive phage mutants were performed at 50°C for 90 min. The lysates were then collected, filtered and used to infect BG214 cells at 30°C to have the effect of the thermosensitive mutation only in the donor, and not in the recipient strain. Mutations in genes *35, 38*, or *39* blocked the transfer of the plasmid with homology (pBG55), with more than 1000-fold reduction in the transduction frequency (**Figure 2A**). A similar result was obtained with *sus109*, bearing a mutation in gene *40* (data not shown). Mutations in the exonuclease (G*34*.*1*P) or in the viral SSB (G*36*P) reduced the transduction frequency by ∼12-fold, whereas the mutation in G*44*P only reduced it by ∼4-fold.

To analyze if these proteins are also involved in the transfer of plasmids having no homology with the SPP1 phage, or just very short homologous regions (sequences of 11–16 bp complementary to SPP1 DNA, see Supplementary Table S1) we performed transduction assays with the natural occurring pUB110 plasmid and the different phage mutants (**Figure 2B**). As already observed the transduction frequency of this plasmid was reduced by a factor of ∼100-fold compared to the frequency of pBG55 transduction. The transduction frequencies were reduced in all of the SPP1 mutants, and similarly to the results obtained with the plasmid having homology, mutations in the recombinase or in replication proteins drastically reduced the phage-mediated transfer of pUB110, whereas mutations in the exonuclease, the SSB, or the HJ resolvase reduced the number of transductants/ml to a lesser extent.

### SPP1 Replication and Recombination Proteins Are Essential for the Generation of Plasmid Concatemeric DNA

**FIGURE 3:**
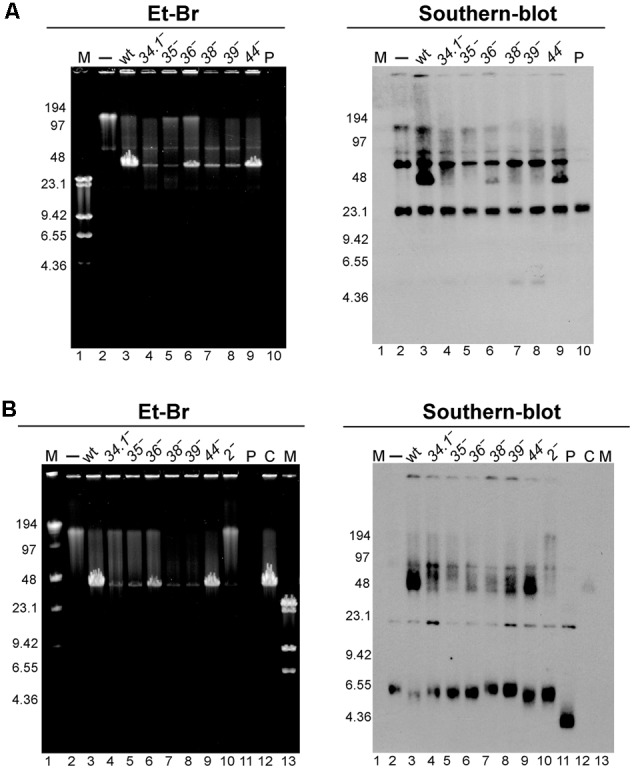
Analysis of the appearance of the transducing particles after infection with different SPP1 mutants of cells bearing RCR replicating plasmids with (pBG55 infections, **A**) or without regions homologous to SPP1 DNA (pUB110 infections, **B**). To unravel the appearance of *hmw* plasmid DNA 30 min after phage infections samples were analyzed by PFGE and Et-Br staining (left panel) followed by Southern-blot (right panel). M, LW and λ-HindIII markers. P, purified plasmid DNA 15 ng (pBG55 in **A**, and pUB110 in **B**); C, control, a SPP1 infection of BG214 cells without plasmid.

Concatemeric plasmid DNA synthesis was observed with RCR plasmids after phage infection (Alonso et al., 1986; Bravo and Alonso, 1990). The results obtained in the previous section suggest that the essential viral recombination (G*35*P) and replication (G*38*P, G*39*P, and G*40*P) proteins could be responsible for the generation of this linear concatemeric plasmid DNA. To test this, we infected BG214 cells bearing pBG55 with the different phage mutants. After 30 min of infection, the infected cells were collected, total DNA was extracted, and separated by PFGE and Southern blotted to detect the production of concatemeric plasmid DNA forms. After infection with the wt phage the appearance of plasmid DNA that migrates with the bulk of SPP1 DNA (i.e., a multimeric plasmid DNA band of 44-kb) was observed (**Figure 3A**). In the absence of G*35*P, G*38*P or G*39*P, the production of this concatemeric band was not observed, consistent with the above result that mutations in these proteins block plasmid transduction. In agreement with its minor role in plasmid transfer, the 44-kb plasmid DNA band was observed after infection with phages bearing mutations in G*34*.*1*P, G*36*P, or in G*44*P. Moreover, the amount of 44-kb pBG55 DNA observed by PFGE and Southern blot correlated in these mutants with their transduction frequencies.

We also observed the appearance of a similar 44-kb plasmid band after infection with the wt SPP1 phage of cells bearing the natural pUB110 plasmid (**Figure 3B**). In concordance with observations using the plasmid with extensive homology, the appearance of this 44-kb plasmid DNA band was clearly observed after infections with SPP1wt and SPP1ΔA phages, which showed the highest transduction frequencies.

### Viral Replication and Recombination Proteins Are Not Involved in the Establishment of the Transduced Plasmid

The results presented above and in earlier reports (Deichelbohrer et al., 1985; Bravo and Alonso, 1990) indicate that a concatemeric ∼44-kb plasmid DNA is encapsidated into the viral capsids. Once this concatemeric plasmid DNA (5.4 plasmid copies in the case of pBG55 plasmid) is injected into a recipient cell, it needs to circularize and monomerize to prepare the plasmid for correct replication and segregation cycles. The duplicated regions present in the concatemer could be used for monomerization, through a homologous recombination event, as it occurs during natural plasmid transformation (Kidane et al., 2009). In order to analyze if the viral replication and recombination machinery is involved in this monomerization and plasmid establishment process, we performed transduction assays with *sup3* as donor and wt as recipient cells (**Table 3**). It appeared that none of the viral proteins were required for the establishment of the transduced plasmid in the recipient cells.

**Table 3:** Viral replication and recombination proteins are not involved in the establishment of transduced plasmids.

Donor strain^a^ and plasmid	Recipient strain	Phage	Transduction Frequency^b^	SD^c^	TF^M^/TF^wt^
BG295 pBG55	BG214	SPP1 wt	4.3 × 10^-3^	±2.0 × 10^-3^	1.00^d^
BG295 pBG55	BG214	*34.1*^-^	3.4 × 10^-3^	±1.5 × 10^-3^	7.9 × 10^-1^
BG295 pBG55	BG214	*35*^-^	2.1 × 10^-3^	±1.1 × 10^-3^	4.9 × 10^-1^
BG295 pBG55	BG214	*36*^-^	3.4 × 10^-3^	±1.8 × 10^-3^	7.9 × 10^-1^
BG214 pBG55^e^	BG214	*38*^-^	4.3 × 10^-3^	±2.1 × 10^-3^	1.0 × 10^0^
BG295 pBG55	BG214	*39*^-^	2.9 × 10^-3^	±2.0 × 10^-3^	6.7 × 10^-1^

*^a^BG214 is the wild type strain and BG295 is the isogenic *sup3* strain. ^b^The transduction frequency (Neo^*R*^/CFU) is the average of at least three independent experiments. ^c^SD: standard deviation. ^d^The frequency of pBG55 plasmid transduction with the phage mutants (TF^*M*^) with respect to the wt phage (TF^*wt*^) is presented. ^e^The *38^-^* mutant is a thermosensitive phage (tsB3), therefore the infection was done at permissive temperature (30°C) and the transduction at non-permissive temperature (50°C) to have the mutation only in the recipient strain.*

### The Influence of Plasmid Copy Number and Replication Mode in Transduction

Plasmid-borne genes are transduced at much higher frequency than chromosomal-borne genes (Ferrari et al., 1978; Deichelbohrer et al., 1985), suggesting that copy number of plasmids could account for such differences. However, there is no tight correlation. As an example, it was published that the transduction frequency of plasmid pUB110, which has ∼50 copies per cell (Viret and Alonso, 1988) is lower than that of pC1943 with ∼15 copies per cell (Deichelbohrer et al., 1985). We confirmed these results (**Table 4**). This suggests that plasmid copy number is not the major determining factor, or not the only one. Other factors such as the presence of pseudo-*pac* sites, or of single-stranded (ssDNA) plasmid forms (recombinogenic particles, see below) could be the cause of this increased transduction frequency. Both plasmids, pUB110 and pC194, are RCR plasmids, but it was found that pC194 is more prone to formation of ssDNA than pUB110 (te Riele et al., 1986; Viret and Alonso, 1987).

**Table 4:** Transduction frequency of theta and rolling circle replicating plasmids without sequence homology with SPP1.

Plasmid	Ab^R^ marker	Replication mechanism^a^	Copy number^b^	ssDNA production^c^	*pseudo*-*pac* site^d^	Transduction Frequency^e^	CI_0.95_^f^
pUB110	Nm	RCR	50	+	–	2.1 × 10^-5^	± 1.3 × 10^-5^
pUB110-cop1	Nm	RCR	15	+	–	4.3 × 10^-6^	±2.2 × 10^-6^
pC194	Cm	RCR	15	+++	–	5.2 × 10^-5^	±4.7 × 10^-5^
pHP13	Cm	RCR	5	+++	–	5.9 × 10^-6^	± 2.9 × 10^-6^
pBT233N	Nm	TR	8	–	–	8.2 × 10^-8^	±7.1 × 10^-8^
pNDH33	Cm	TR	6	–	1	6.4 × 10^-7^	±3.2 × 10^-7^

*^a^RCR, rolling circle replication; TR, theta replication. ^b^Plasmid copy numbers were reported in the literature and are presented here for comparison. ^c^ssDNA production was reported in the literature and is presented here for comparison. ^d^The *pac* motif (5′-CTATTGCGG⇓C-3′) is absent in all of the plasmids. Here the presence of a shorter motif that we call *pseudo-pac* site 5′-TTGCGG⇓CW-3′ is indicated. ^e^The transduction frequency (transductans/CFU) is the mean of at least five independent experiments. ^f^CI, confidence interval.*

To elucidate the influence of copy number, we compared the transduction efficiency of plasmid pUB110 (48 ± 4 copies/cell) and its derivative pUB110-cop1 (9 ± 1 copies/cell). pUB110-cop1 results from a single mutation in pUB110 plasmid, and therefore it has the same amount of ssDNA as the parental plasmid, but its copy number is reduced by 5-fold (Leonhardt, 1990). Both plasmids should have similar rates of circularization and establishment when they are injected into the recipient cell. As shown in **Table 4**, the transduction efficiency of pUB110-cop1 was proportionally reduced 4.6 times. In parallel we compared also the transduction frequencies of two other plasmids that accumulate ssDNA, pC194 (15 ± 2 copies per cell, Alonso and Trautner, 1985) and pHP13 (a pTA1060 derivative, 7 ± 2 copies per cell, Wang et al., 2004). Here also the transduction efficiency decreased by lowering the copy number of the plasmids. Nevertheless in all cases the transduction frequencies were higher for the plasmids accumulating ssDNA intermediates (**Table 4**).

**FIGURE 4:**
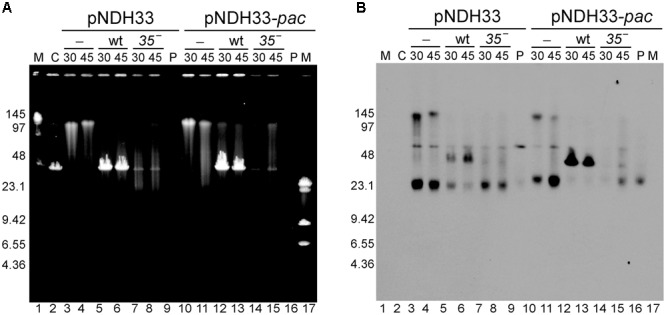
Southern-blot analysis of the appearance of the transducing particles after infection with SPP1 or with *sus35* phage of cells bearing TR plasmids having (pNHD33-*pac*) or lacking (pNDH33) homologous regions to the phage. **(A)** Ethidium bromide stain and **(B)** Southern blot of the same gel developed with a chloramphenicol probe to visualize plasmid DNA. Lanes: 1 and 17: LW and λ-HindIII markers. Lane 2: C, control SPP1 infection of BG214 cells without plasmid. Lanes 3–4 and 10–11: control, non-infected BG214 cells bearing pNDH33 or pNDH33-*pac* plasmid. Lanes 5–6 and 12–13: SPP1 infection of BG214 cells bearing pNDH33 or pNDH33-*pac* plasmid, after 30 and 45 min infection. Lanes 7–8 and 14–15: BG214 cells bearing pNDH33 or pNDH33-*pac*, after 30 and 45 min infection with *sus35* phage. Lane 9 and lane 16: P, 15 ng of purified pNDH33 or pNDH33-*pac* respectively.

Previous studies of the plasmid transduction by the SPP1 phage were done only with RCR plasmids. To determine the transduction frequency of theta replicating (TR) plasmids we used two such plasmids: pBT233 and pNDH33, which have a copy number similar to that of pHP13 plasmid (**Table 1**). Plasmid pBT233 is a pSM19035 derivative (erythromycin resistant), which has a copy number of ∼8 ± 2, and replicates unidirectionally by a DNA polymerase I (PolI)-dependent theta mechanism (Ceglowski et al., 1993a,b,c). Plasmid pNDH33 is a derivative of pBS72 (chloramphenicol resistant) with a copy number of ∼6 ± 1 plasmids/cell (Nguyen et al., 2005; Phan et al., 2006). pNDH33 is thought to replicate by a DnaA-dependent and DNA PolI-independent theta type mechanism (Titok et al., 2003; Schumann, 2007). To compare TR and RCR plasmids, and to eliminate any resistance marker effects, the neomycin gene of the pUB110 was cloned into plasmid pBT233, to render plasmid pBT233N. The transduction frequency of the TR plasmid pBT233N was about 70-fold lower than that of pHP13. We measured also the transduction frequency of the second TR plasmid, pNDH33. This appeared to be also low, but only ∼10-fold lower than that of pHP13 plasmid (**Table 4**). This higher transduction could be due to the occasional presence in the pNDH33 plasmid of a *pseudo-pac* site or because of a 16 bp stretch of homology (**Table 4** and Supplementary Table S1). When analyzing the fate of TR plasmids in infected cells, it was observed that, as with RCR plasmids, the infection with wt SPP1 phage produced the accumulation of a 44-kb plasmid DNA band, which was not observed after infection with a *sus35* mutant (**Figure 4**).

### The Presence of Homology to Phage Enhances the Transduction of TR Plasmids

When the phage packaging signal (*pac*) was integrated into the host chromosome, SPP1 mediated the transduction of chromosomal genes located close to the region of integration of the *pac* signal (Bravo et al., 1990). It was not tested if the presence of other SPP1 regions also increases the transduction frequencies of chromosomal DNA. To test this, we used the pBT233N derivative conferring Nm^R^, which replicates via the theta-type mechanism as the chromosome. Different regions of SPP1 were cloned into pBT233N in order to evaluate whether the presence of *pac* sequence or the replication origin (*ori*L) results in higher transduction than simply homology to the phage (**Table 5**). Overall, the presence of a homologous region increased the transduction frequency of pBT233N plasmid by more than 1000-fold, and this increase was observed independently of the homologous region cloned (*pac, ori*L, or a 400 bp or 1000 bp region unrelated to replication and packaging processes). Similarly, cloning into a RCR plasmid (pHP13) one of the phage origins of replication of SPP1 did not further increase the transduction frequency (Supplementary Table S2). Using of other TR-type replicon, pNDH33, provided similar results (**Table 5**). Furthermore, the accumulation of the 44-kb plasmid band was higher in the TR plasmids derivatives having homology with the phage (**Figure 4**, and data not shown).

**Table 5:** Transduction frequency of theta replicating plasmids bearing different SPP1 DNA regions.

Plasmid	Length of homologous region	Special features	Transduction Frequency^a^	CI_0.95_^b^
pBT233N	–	–	8.2 × 10^-8^	±7.1 × 10^-8^
pBT233N-400	414 bp	homology	3.1 × 10^-5^	±1.9 × 10^-5^
pBT233N-1300	1340 bp	homology	4.5 × 10^-4^	±3.0 × 10^-4^
pBT233N-*pac*	412 bp	*pac*	1.7 x 10^-4^	±2.8 × 10^-4^
pBT233N-*ori*L	360 bp	*ori*L	1.5 x 10^-4^	±1.6 × 10^-4^
pNDH33	–	–	5.6 × 10^-7^	±2.9 × 10^-7^
pNDH33-1300	1340 bp	homology	2.0 × 10^-4^	±8.5 × 10^-5^
pNDH33-*pac*	412 bp	*pac*	3.9 x 10^-4^	±3.2 × 10^-4^

*^a^The transduction frequency (transductans/CFU) is the mean of at least five independent experiments. ^b^CI, confidence interval.*

## Discussion

Until recently, it was thought that generalized transduction occurred at low frequency. However, recent single-cell analyses observed transduction rates close to 1% per plaque forming units when natural communities were used as recipients (Kenzaka et al., 2010). Therefore the study of the transduction mechanisms is essential to prevent this highly frequent horizontal gene transfer process, to avoid the spread of antibiotic resistance among bacteria. In this aspect, the SPP1 bacteriophage is a valuable model, because its replication, recombination, and packaging machineries haven been studied in deep for many years. Furthermore, it was recently reported that SPP1 can occasionally infect resistant cells when combined with sensitive cells, providing new routes for horizontal gene transfer (Tzipilevich et al., 2017). Previous biochemical studies assigned a role to SPP1 proteins G*34*.*1*P, G*35*P, G*36*P, G*38*P, G*39*P, G*40*P, and G*44*P in replication and recombination, but their contribution to generalized plasmid transduction remained unknown. Here we show that all SPP1 replication proteins contribute to horizontal plasmid transfer, although to a different extent. The origin binding protein (G*38*P), helicase loader (G*39*P), and helicase (G*40P*) are essential to produce concatemeric plasmid DNA, which is synthesized after phage infection. Infections with the *sus36* mutants show only a 10-fold reduction in the transduction frequency, probably due to potential complementation of the G*36*P function by cellular SsbA protein (Seco et al., 2013; Seco and Ayora, 2017). The SPP1 recombination proteins contribute to plasmid transfer to a different extent. The exonuclease G*34*.*1*P and the Holliday junction resolvase G*44*P only contribute partially to plasmid transduction, with a reduction of the transduction frequency of 12- and 5-fold in their mutants, respectively. The G*35*P recombinase is essential, with its inactivation leading to a >100-fold decrease.

Previous studies with SPP1 and RCR plasmids showed that: (i) the transduction of pUB110 and pC194 plasmids was enhanced 100- to 1000-fold when there was any homology between the plasmid and the SPP1 genome rather than with the specific *pac* signal; (ii) pUB110 and pC194 plasmid transduction was independent on RecA (Deichelbohrer et al., 1985), and (iii) linear plasmid concatemeric DNA (or high-molecular-weight [*hmw*] DNA) accumulated during phage infection, and in certain genetic backgrounds (Viret and Alonso, 1987; Viret et al., 1991). The synthesis of *hmw* DNA and its independence of the host-encoded recombinase (RecA) strongly suggests that the formation of transducing particles may rely on viral replication and/or recombination functions. In this work we show that the synthesis of this *hmw* DNA, and consequently transduction of RCR plasmids requires an active G*35*P protein. Biochemical analysis shows that G*35*P is an ATP-independent single-strand annealing enzyme, similar to the RecT enzyme encoded by the Rac prophage (Ayora et al., 2002). Both, G*35*P and RecT, belong to the Redβ family of viral single strand annealing proteins. To date, five different single strand annealing recombinase families have been identified in phages: Sak, Redβ, Erf, Sak4 and Gp2.5 (Lopes et al., 2010). These recombinases have gained increased attention in recent years because of their abundance in phage genomes (Lopes et al., 2010; Delattre et al., 2016), and also due to their wide use in recombineering systems (Datta et al., 2008; Sun et al., 2015). Many of these recombinases, including G*35*P, are essential for the phage life cycle (Zecchi et al., 2012; Neamah et al., 2017).

In this work we found that variations in copy-number affect the transduction frequency. Since the transduction is a stochastic process, it is expected that the more plasmid DNA in the cell the more generalized transducing phage particles should carry a plasmid copy and therefore the chances of transduction increase. The plasmids replicating in *B. subtilis* cells are either of the TR (circle-to-circle) type or RCR (sigma) type, and the products of both replication modes are usually covalently closed circular monomers (Khan, 2005). Comparing plasmids with similar copy number we observed that the frequency of transduction for RCR plasmids is ∼60-fold higher than that for TR plasmids. This result suggests that the type of DNA replication also determines the transduction frequency. In the small RCR plasmids leading and lagging strand replication are uncoupled, and they contain two modules: the Rep protein with its cognate double-strand origin (DSO), and a single strand origin (SSO), which functions as the major initiation site for lagging-strand synthesis (Alonso et al., 1988; Espinosa et al., 1995; Khan, 2005). All RCR plasmids accumulate ssDNA although to a different extent: pUB110 accumulates traces and pC194 accumulates circular ssDNA (te Riele et al., 1986; Viret and Alonso, 1988). In contrast, the large low-copy-number TR plasmids, such as pBT233, which replicates via an unidirectional mechanism, do not accumulate circular ssDNA intermediates (Ceglowski et al., 1993b,c). We propose that the high transfer frequencies of some RCR plasmids may be correlated with the high accumulation of recombinogenic ssDNA intermediates in these plasmids. Such ssDNA intermediates may constitute the substrates for formation of the transducing particles, through a recombination catalyzed by the G*35*P protein. This is in agreement with recent results observed with viral recombinases: when analyzing their recombineering activity *in vivo*, it was found that they catalyze single-strand annealing preferentially on the lagging strand (van Kessel and Hatfull, 2008; Mosberg et al., 2010; Lajoie et al., 2012; Fricker and Peters, 2014; Ander et al., 2015). We propose that all the phages encoding recombinases will transduce RCR plasmids with high efficiency by the mechanism of viral recombinase-mediated generalized transduction. Furthermore, we also observed that the transduction of the pUB110 and pNDH33 plasmids, which do not have an extensive region of homology, was strongly reduced in infections with the *sus35* mutant (**Figures 2, 4**). All phage recombinases studied so far are single-strand annealing proteins that promote genetic recombination under more permissive conditions than RecA (Scaltriti et al., 2011; De Paepe et al., 2014; Menouni et al., 2015). Our results suggest that G*35*P contributes to the transfer of natural plasmids by catalyzing a recombination reaction using small stretches of homology found in many plasmids (Supplementary Table S1).

The different contributions of the SPP1 recombination proteins to plasmid transduction, together with the high recombinogenic nature of the RCR plasmids, suggest that the initial DNA substrate, used for the production of transducing particles by recombination, is indeed ssDNA. This is consistent with the result that the G*34*.*1*P exonuclease, which resects the dsDNA ends to generate the appropriate substrate for the recombinase (Martinez-Jimenez et al., 2005), has a minor role in plasmid transfer. Similarly, we found that the SPP1 SSB protein, G*36*P, only slightly contributes to the mechanisms of plasmid transduction. However, in some phages the recombinases require the activity of their cognate SSB proteins to perform their function (Neamah et al., 2017).

It was previously observed with RCR plasmids that any SPP1 DNA segment larger than 50 bp, cloned into such plasmids, greatly increased the transduction frequency (Deichelbohrer et al., 1985; Alonso et al., 1986). We extend this observation to TR plasmids, where the transduction frequency was highly increased, independently of what is the region of homology cloned, whether it was the packaging sequence, a phage origin of replication, or any other region of homology. Similarly, the cloning of the origin of replication of SPP1 (*ori*L*)* into a RCR-type plasmid did not further increase its transduction frequency (Supplementary Table S2). We conclude that any DNA region homologous to the phage genome increases the frequency of horizontal transfer of plasmids, independently of their replication mechanism. Enhanced transduction of plasmids bearing homology with phage DNA has been also observed with phage T4, which codes for a different recombinase, the UvsX protein (Kreuzer et al., 1988), and with *Salmonella typhimurium* phage P22, which codes for the Erf recombinase (Orbach and Jackson, 1982).

How is the plasmid substrate for generalized transduction generated? Three different mechanisms could account for the generation of a concatemeric plasmid DNA with high frequency of transduction. In the first model, the multiple tandem repeats of plasmid DNA might be produced by intermolecular recombination, as proposed for P22 plasmid transduction (Mann and Slauch, 1997). This mechanism resembles phage T4 generation of concatemeric DNA during its replication (Kreuzer, 2000; Mosig et al., 2001). Here, multiple strand invasions catalyzed by the ATP-dependent RecA-like recombinase encoded by this phage, UvsX, and the resolution of the Holliday junction intermediates by its Holliday junction resolvase Gp49 (also called EndoVII), produce the concatemeric DNA, as well as the transducing particle (Kreuzer et al., 1988; Kreuzer, 2000; Mosig et al., 2001). We do not favor this hypothesis in the SPP1 system, because we found that the Holliday junction resolvase G*44*P has only a minor role in plasmid pBG55 and pUB110 transduction. In the second model, plasmid over-replication leads to the accumulation of linear concatemeric *hmw* DNA (Cohen and Clark, 1986; Viret and Alonso, 1987; Viret et al., 1991). The accumulation of linear head-to-tail multigenome-length plasmid DNA (*hmw* DNA) in the absence of RecBCD/AddAB was documented in both *Escherichia coli* and *B. subtilis* cells (Silberstein and Cohen, 1987; Viret and Alonso, 1987). Indeed, upon infection, many bacteriophages directly or indirectly inactivate end-resection catalyzed by this host encoded multi-subunit helicase-nuclease enzyme (Szczepanska, 2009). It was observed that the synthesis of pC194 or pUB110 *hmw* plasmid DNA occurred in the absence of plasmid-encoded Rep protein, and required DNA PolI, RecA and pre-primosomal proteins (e.g., DnaB) (Viret and Alonso, 1987; Leonhardt et al., 1991; Viret et al., 1991). Analysis of this *hmw* plasmid DNA by electron microscopy displayed linear DNA molecules up to 100 kb in size, which were either single-stranded, double-stranded or duplex DNA with single-stranded tailed ends (Leonhardt et al., 1991). This *hmw* DNA can be encapsidated into a viral prohead by a headful packaging mechanism (Schmidt and Schmieger, 1984; Schmieger, 1984). If this model is correct, the presence of a *pac* signal will significantly increase the encapsidation of the plasmid *hmw* DNA, and we found that there was not an increase in the transduction frequency when plasmids contained the *pac* signal. In the third model, phage infection arrests host and plasmid replication. Then SPP1-dependent replication restarts, and the linear plasmid concatemer is synthesized. This is consistent with the result that the phage G*38*P protein may act as a PriA-like enzyme, restarting DNA replication outside form a replication origin (Seco et al., 2013; Seco and Ayora, 2017). In this *de novo* synthesis of plasmid DNA, a viral *pac* site might be gained by recombination and recognized by the viral packaging machinery (Alonso et al., 1986; Bravo et al., 1990; Viret et al., 1991). In this model, the phage might form a phage-plasmid chimera and the plasmid hijacks the viral replication machinery to promote *de novo* synthesis of linear plasmid concatemeric DNA. The concatemeric plasmid DNA is then packaged into an empty prohead by the headful mechanism, indistinguishable of viral DNA, provided that the packaged substrate is larger than mature phage DNA. Our data support the third model, because we found that in infections with a phage bearing a mutation in the terminase (*sus19* infections), plasmid concatemers up to 200-kb long are produced after phage infection (**Figure 3B** and Supplementary Figure S2). This model explains also the requirement of viral replication proteins for the formation of the transducing particles. However, we were unable to detect the phage-plasmid chimeras, which might be rapidly processed to produce the plasmid head-to-tail concatemers.

Our results show that the establishment of the transduced concatemeric plasmid in the host is independent of phage encoded recombination functions, which only participate in the generation of the transducing particle. We propose that the injected linear concatemer can be converted into a circular form by the homologous recombination machinery of the recipient cells. In this respect, transduction of plasmids might have similar host requirements as the resolution of phage-plasmid chimeras analyzed in the P22 and SPP1 systems (Orbach and Jackson, 1982; Alonso et al., 1992). In the former case, the plasmid integrated into the phage genome has to be excised from the genome of the defective phage prior to establishment, whereas in the latter case the head-to-tail plasmid concatemer has to recombine intramolecularly to facilitate plasmid establishment. This process was found to be RecA-independent but dependent on host RecO and RecR functions that also catalyze single-strand annealing (Alonso et al., 1992; Manfredi et al., 2008).

## Author Contributions

AV-R, ML-S, AQ-O, and SA: performed the experiments; AV-R, AS, and SA: analyzed data; SA: conceived the project, integrated the results and wrote the paper.

## Conflict of Interest Statement

The authors declare that the research was conducted in the absence of any commercial or financial relationships that could be construed as a potential conflict of interest.

## Abbreviations

PFGE: pulsed field gel electrophoresis
RCR: rolling circle replication
SPP1: *B. subtilis* bacteriophage SPP1
*sus*: suppressor sensitive (mutation)
TR: theta replication
ts: thermosensitive
wt: wild type
