# Supplementary material for: Molecular Mechanisms That Contribute to Horizontal Transfer of Plasmids by the Bacteriophage SPP1
Source: Front Microbiol. 2017 Sep 22;8:1816. doi: 10.3389/fmicb.2017.01816 (PMC5615212; doi:10.3389/fmicb.2017.01816)
Supplement: Supplementary file 1 [file Data_Sheet_1.PDF]

Supplementary Material

Molecular mechanisms that contribute to horizontal transfer of plasmids by the bacteriophage SPP1

Ana Valero-Rello[1,2,3], María López-Sanz[1,3], Alvaro Quevedo-Olmos[1], Alexei Sorokin[2], and Silvia Ayora[1,*]

*** Correspondence:** Silvia Ayora, e-mail: sayora@cnb.csic.es.

1. Supplementary Tables

Table S1: Short stretches of homology with SPP1 DNA found in the plasmids used in this study

Table S2: Transduction frequency of the RCR replicating plasmid pHP13, and its derivatives bearing different SPP1 DNA regions

2. Supplementary Figures

Figure S1: Effect of the *34.1* mutation on phage plaque size

Figure S2: Accumulation of *hmw* plasmid DNA after phage infections

Table S1. Short stretches of homology with SPP1 DNA found in the plasmids used in this study.

Plasmid	Homology length	Plasmid coordinates
pUB110	13	891 – 903
	14	219 – 232
		2288 – 2301
		3133 – 3143
		3398 – 3411
pC194	13	468 – 480
		485 – 497
		2256 – 2268
		2775 – 2787
	14	247 – 260
		1580 – 1593
	15	2039 – 2053
		2779 - 2793
pHP13	11	4286 – 4303
	12	4367 – 4378
	13	3252 - 3264
	14	2480 - 2493
pBT233N	11	3386 – 3396
		9383 – 9393
	12	9288 – 9299
	13	271 – 283
		7164 – 7176
	14	1498 – 1511
		2758 – 2771
		6327 – 6340
		7246 – 7259
		8030 – 8045
		8877 – 8890
	15	3528 – 3842
pNDH33	11	5379-5389
	12	5285-5296
	14	2660-2673
		2165-2178
	16	5000-5015

Table S2. Transduction frequency of the RCR replicating plasmid pHP13, and its derivatives bearing different SPP1 DNA regions

**Plasmid**	**SPP1 DNA cloned**[a]	**Length of homologous region (bp)**	**Special Features**	**Transduction Frequency**[b]	**$CI_{0.95}$**[b]
pHP13	–	–	-	5.9×10^{-6}	$\pm 2.9 \times 10^{-6}$
pBT400	3225-4089	864	homology	4.1×10^{-4}	$\pm 2.9 \times 10^{-4}$
pBT271	33875-36850	2975	*ori*L	1.6×10^{-3}	$\pm 7.9 \times 10^{-4}$
pBT163	43778-2439	2675	*pac*	1.1×10^{-3}	$\pm 3.7 \times 10^{-4}$

[a]The SPP1 coordinates that where cloned are indicated

[b]The transduction frequency (transductans/CFU) is the mean of at least five independent experiments.

[b]CI: confidence interval

Supplementary Figure S1

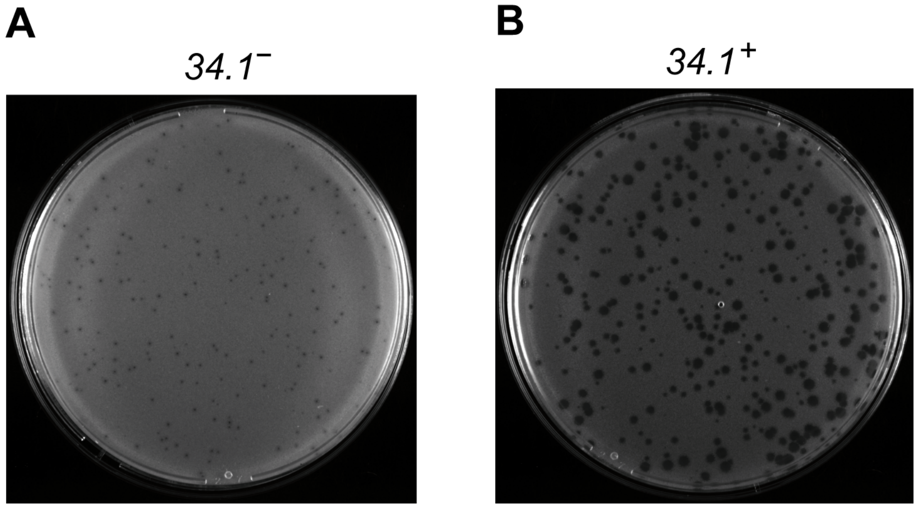

Figure S1. Effect of the *34.1* mutation on phage plaque size. Approximately $5x10^7$ bacteria were infected with 300 p.f.u. of the *sus34.1* phage, and plated on LB-Mg agar. Plates were incubated 24h at 37 °C and photographed. (**A**) BG214 (*wt*) infection. (**B**) BG295 (*sup3*) infection.

Supplementary Figure S2

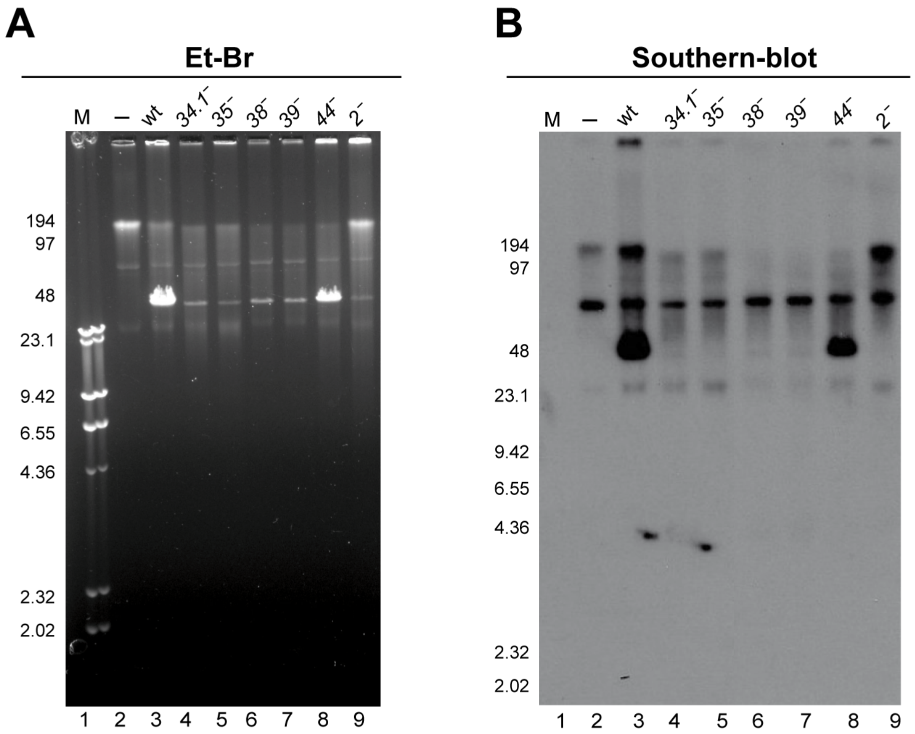

Figure S2: Accumulation of *hmw* plasmid DNA after phage infections. Analysis by PFGE and Et-Br staining (left panel) and Southern-blot (right panel) of the appearance of *hmw* pBG55 plasmid DNA 30 min after phage infections. Lane 1: λ DNA-HindIII marker, Lane 2: no phage; lane 3: SPP1 wt infection; lane 4: *sus34.1* (*34.1*$^-$), lane 5: *sus35* (*35*$^-$), lane 6: tsB3 (*38*$^-$), lane 7: *sus53* (*39*$^-$), lane 8: SPP1ΔA (*44*$^-$), and lane 9: *sus19* (*2*$^-$) infection.
